# Chemical constituents and anti-ulcer effects of a wild pear (*Pyrus syriaca* Boiss.): Phytochemical, histopathological and apoptotic approaches

**DOI:** 10.1371/journal.pone.0344660

**Published:** 2026-04-02

**Authors:** Khaled Abdul-aziz Ahmed, Shatha Nasih Tawfeeq, Sheylan Salah Abdullah, Goran Noori Saleh, Ahmed Aj. Jabbar, Abdulmohsen I. Algefare, Manal A. Alfwuaires, Muzhda Haydar Saber, Mustafa Abdul-Monam Zainel, Talal Salem Al-Qaisi, Ahlam Dhafer Alshehri, Hanan Ibrahim Alsharif, Khalid M. Alqaisi, Mohamad Fawzi Mahomoodally

**Affiliations:** 1 Department of Basic Dental Sciences, Faculty of Dentistry, Al-Ahliyya Amman University, Amman, Jordan; 2 Basic Science Department, College of Dentistry, Tikrit University, Tikrit, Iraq; 3 Department of Medical Laboratory Technology, Erbil Technical Health and Medical College, Erbil Polytechnic University, Erbil, Iraq; 4 Department of Nursing, Tishk International University, Erbil, Iraq; 5 Department of Biological Sciences, College of Science, King Faisal University, Al-Ahsa, Saudi Arabia; 6 Department of Nursing, Lebanese French University, Erbil, Iraq; 7 Department of Pharmacology, College of Pharmacy, Knowledge University, Kirkuk Road, Erbil, Iraq; 8 Department of Biomedical Sciences, College of Health Sciences, Abu Dhabi University, Abu Dhabi, United Arab Emirates; 9 Department of Chemistry, College of Science, University of Jeddah, Jeddah, Saudi Arabia; 10 Department of Medical and Clinical Laboratory Technology, Faculty of Allied Medical Sciences, Applied Science Private University (ASU), Amman, Jordan; 11 Institute of Research and Development, Duy Tan University, Da Nang, Vietnam; 12 School of Engineering & Technology, Duy Tan University, Da Nang, Vietnam; 13 Department of Biochemistry, Saveetha Medical College and Hospital, Saveetha Institute of Medical and Technical Sciences, Chennai, Tamil Nadu, India; 14 Centre of Excellence for Pharmaceutical Sciences (Pharmacen), North West University, Potchefstroom 2520, South Africa; 15 Department of Health Sciences, Faculty of Medicine and Health Sciences, Reduit, University of Mauritius, Mauritius; National University of Medical Sciences, PAKISTAN

## Abstract

Gastric ulcers are a major health concern that can cause severe complications such as bleeding (perforation), digestive blockage, or even stomach cancer if not treated properly. In this study, we evaluated the phytoconstituents, acute toxicity, and gastroprotective potential of the unexplored *Pyrus syriaca* Boiss. in ethanol-mediated gastric ulcer in rats. Experimental rats were housed in different cages and fasted before receiving pretreatments (250 and 500 mg/kg) and absolute ethanol (5 mL/kg, i.g.) administration. Phytochemical investigations unveiled increased phenolic (142.55 GAE/100 g), flavonoid (73.65 QE/100 g), and anthocyanins (44.05 CGE/100 g) in methanolic extracts of *Pyrus syriaca* (MEPS). The toxicity evaluation of MEPS (2 and 5 g/kg) did not show any behavioral or physiological changes in rats, even after 14 days of trial. The *in vivo* gastroprotective evaluation of MEPS (250 and 500 mg/kg) showed a dose-related protection against ethanol-mediated ulceration comparable to lansoprazole. MEPS pretreatment (250 and 500 mg/kg) improved gastric defense barriers (mucus content and pH maintenance) as well as ameliorated gastric tissue alterations, lowering ulcer index areas by 22.13% and 50.40%, respectively. Interestingly, MEPS exhibited significant antioxidant and anti-inflammatory potentials, denoted by up-regulated superoxide dismutase, catalase, interleukin (IL-10), while lowering MDA, TNF-α, and IL-6 cytokines. The anti-apoptotic effects of MEPS were confirmed by down-regulated Bax (by 35.29%, and 47.05%, respectively) and increased Bcl-2 expressions (by 51.42% and 60%, respectively). Collectively, *P. syrica* showed enriched phytoconstituents that supported its gastroprotective potentials through modulation of antioxidant, inflammatory, and apoptotic cascades.

## 1. Introduction

Gastric ulcer (GU) is the most pervasive digestive disorder worldwide, being notorious in underdeveloped nations due to poor sanitation, *H. pylori* infections, as well as limited access to pure water/proper healthcare. Statisticians confirmed that more than 5–10% of individuals will acquire GU at some point in their lifetime [1], while the global annual incidence rates were estimated as 0.3–1.9% [2]. Over the years, numerous etiologies have been highlighted as contributors to gastric ulcer development, such as helicobacter infection, psychological factors, smoking, stress, NSAIDs, and alcohol consumption. Heavy alcoholism has been a major risk factor associated with the initiation of gastrointestinal erosion/bleeding [3]. Continuous exposure of the stomach to aggressive factors, such as alcohol, weakens gastric defense barriers (reduced mucus/mucopolysaccharides and lowers gastric pH) that ultimately damage the epithelial layer and cause mucosal injury as well as submucosal edema. The gastric physical barriers, blood flow, prostaglandins, as well as mucus/bicarbonate secretions, are considered key factors essential for maintaining mucosal integrity [4].

Alcohol can provoke both acute and chronic gastric mucosal injury, each of which causes up-regulated production of gastric acids. The stomach acid up-regulation includes up-regulation of histamine for H_2_R activation, acetylcholine for M_3_R activation, and gastric for CCK_2_R. Once H2R is activated, several reaction cascade begins that subsequently provoke H^+^/K^+^ ATPase in parietal cells, which leads to increased hydrochloric acid production and gastric mucosal damage [5]. Heavy alcoholism also causes cellular and systemic toxicity through up-regulation of acetaldehyde and ROS molecules, which alters cellular process that subsequently initiates oxidative stress and inflammation (by altering gastric permeability/microbiomes and endotoxin production), and mitochondrial damage. The reaction cascade weakens cell integrity and facilitates the development of alcohol-associated disease through various tissue/organ damage [6]. Pharmaceutical innovations have proposed numerous chemical synthetics for GA, most of which are histamine H_2,_ such as omeprazole and lansoprazole, for managing gastritis; however, they have only limited therapeutic efficacy, and numerous adverse effects related to tolerability and relapse. Therefore, researchers are continuously investigating to provide more efficient and safer alternatives to chemical synthetics for managing gastric disorders [7].

Therapeutic plants and their isolated compounds have been consumed as preventive or curative agents by nearly 75% of worldwide nations, according to World Health Organization data [8]. Herbal-based therapeutics are employed as predominant curatives for gastric-related disorders by more than 10% of the population across the globe [9].

### Pyrus syriaca Boiss

It is a well-known fruit-bearing plant that is native to the environments of Iran, Iraq, Syria, and Turkey. The plant grows in open forests/Rocky Mountains as well as humid and semi-arid regions. Its high resistance to drought, frost, and more grafting compatibility made the species a unique choice for the industrial products, including chessboards to decorate wooden boxes [10]. Ethnobotanical studies have shown numerous health benefits of *P. syriaca* fruits (wild pear), which are served as an infusion for cough/ catarrhs and stomach-ache in Iraq [11], or freshly consumed for improving digestion, weight loss, and prepared as a diuretic/laxative syrup in Syria [12]. The fruits commonly known as Adi armut in Turkey, where it is prepared as an expectorant infusion for alleviating stomachaches [13]. Biological investigations have shown numerous bioactivities of *P. syriaca* fruits, including antioxidant [14] and anti-microbial activities [15]. Despite its traditional use and biological potentials, the chemical composition of this wild pear is uncertain, except for its essential oil composition, highlighting linoleic acid, tocopherols, and oleic acid as predominant compounds [14]. In an attempt to unveil the chemical constituents of *P. syriaca* fruits, spectrophotometric assays were employed to evaluate the total phenolic, flavonoid, and anthocyanin contents. This was followed by the acute toxicity and the gastroprotective evaluations in ethanol-mediated gastric ulceration, with depicted molecular mechanisms using histopathological and immunohistochemical assays.

## 2. Materials and methods

### 2.1 Plant collection and extract

The wild Pear fruits were collected from Hujran, Erbil, Iraq (altitude: 36.410076, latitude: 44.263589). After the plant identification by Prof. Dr. Abdullah Sh. Sardar, the voucher number (5403) was taken from the herbarium unit of Salahaddin University. The fruits were dried in the shade, and then they were ground into a fine powder using a mill grinder. An amount of 100 g of dried powder was placed into two flasks containing methanol and ethanol solvents (95% concentration), which were left on a magnetic stirrer overnight (3X). After filtration of the solution mixture (100 mm pore size), the solvents were completely removed using a rotary evaporator. The dried extract (yield 15.5% and 12.9%, respectively) was stored in dark vials (−20 °C) until later use. Since the methanolic extract showed the highest yield, which has been highlighted as a more efficient solvent for polar phytoconstituents, it was used for *in vivo* studies.as a more efficient solvent for polar phytoconstituents, it was used for in vivo studies.

### 2.2 Phytochemical analysis

The methanolic fruit extracts were analyzed for the amount of total phenolic (TF) and flavonoid contents (FC) by applying the Folin–Ciocalteu redox and aluminum trichloride (AlCl_3_) assay, respectively, as previously explained [16]. The anthocyanin content (AC) of MEPS was determined following previous protocols [17] and the results of TF, FC, and AC are expressed as GAE/100 g, quercetin equivalent/100 g, and cyanidin-3-glucoside chloride equivalents (CGE)/100 g of MEPS.

### 2.3 Chemicals and kits

The Eliza kits for antioxidant enzymes, including SOD (Cat. S2147), CAT (Cat. C0979), and PGE2 (Cat. SEKR-0053) were purchased from Sigma Aldrich (Merck, Germany). The tumor necrosis factor-α (TNF-α) (Cat. SEKR-0009); interleukin (IL)-6 (Cat. SEKR-0005); and IL-10 (Cat. SEKR-0006) were obtained from Solarbio Co. (Wuhan, China).

### 2.4 Animals and ethics

Sprague Dawley adult rats from both genders, aged 8–9 weeks and weighing 180–200 g, were purchased from the research center of Erbil Polytechnic University. The study confirms that all experiments were performed in accordance with relevant guidelines and regulations (**ARRIVE** guidelines) [18]. The experimental protocols were approved by the ethics committee of Erbil Polytechnic University (No. 293, 3/9/2025).

### 2.5 Acute toxicity test

The current study considered the safety evaluation of MEPS before using it in repeated doses in a gastroprotective trial. Thirty-six rats from both genders were aligned in three mesh-wired cages (12 rats each), and they were fasted overnight before oral delivery of physiological saline (group A), 2 g/kg MEPS, or 5 g/kg MEPS (B and E, respectively). After treatments, rats were fasted (provided with water) for another 3 hours to allow enough time for the extract absorption. Rats were kept under observation for any possible toxic incidence or behavioral/physiological changes (salivation, skin biting, skin/eye color, diarrhea, tremors, sleep irregularity, or lethargy) or mortality. After two weeks, rats received an intraperitoneal anesthesia injection (1 mL) prepared from 3 mg/kg xylazine and 30 mg/kg ketamine and were humanely sacrificed. The liver and kidneys were collected from rats for histopathological changes, and the centrifuged blood samples were screened for relevant biochemicals [19].

### 2.6 Gastroprotective experiment

#### 2.6.1 Experimental design.

**Fig 1:**
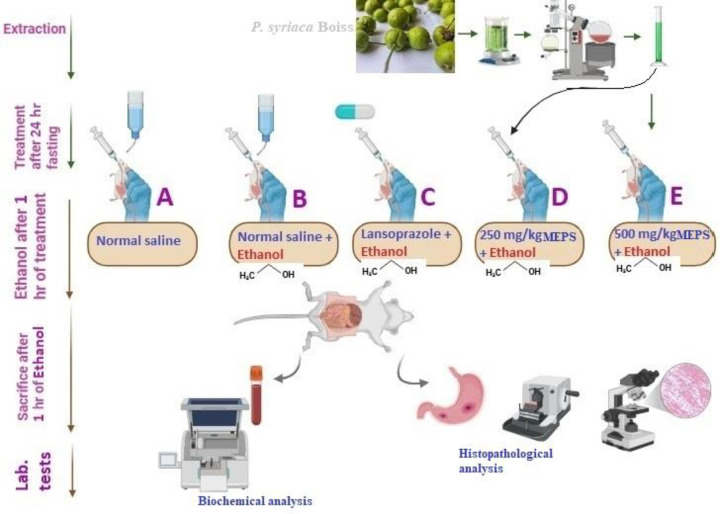
Experimental design for gastroprotective evaluation of MEPS.

Thirty Sprague male rats were assigned to 5 groups (6 rats each), applying a computer-based randomization list to ensure unbiased placement (Fig 1). The animal procedures were conducted by researchers blinded to the group assignments. Rats were fasted for 24 hours and pretreated either with normal saline (groups A and B); 30 mg/kg lansoprazole (group C); or 250 or 500 mg/kg MEPS (groups D and E, respectively). One hour later, groups B-E rats received an oral dosage of absolute ethanol to induce ulceration. Another hour later, rats received intraperitoneal anesthesia injection containing 3 mg/kg xylazine and 30 mg/kg ketamine, and the blood samples were collected by cardiac puncture before euthanasia. The dissected stomach and blood samples were analyzed for relevant histological and biochemical analysis [20].

#### 2.6.2 Gross and ulcer score study.

The gross images of the stomachs were captured after opening at the greater curvature, deepened in 10% formaldehyde for 30 min, and flattened against ice packs. The mucin/mucus content of each stomach was collected before saline washing and gross capturing views. The gastric ulcer/lesions appeared as thick red/dark lines extended across the stomach surface area.

The ulcer scores/index were calculated for each stomach sample using an arbitrary scale, as detailed by previous researchers [21]. Briefly, normal stomach areas were given 0 score; areas with hyperemia scored as 0.5–1; areas with hemorrhagic spots scored as 1–2; small ulcer areas scored as 2–3; several small ulcers scored as 3–4; 1–5 small and 1–3 large ulcers scored as 4–5; several large ulcers scored as 5–6; enlarged ulcers and perforations scored as 6. The scores of ulcer controls were used as positive standards to determine the protective percentage of treated groups using Eq. (1):


$$\text{Protective percentage }(\%)=\;\frac{untreated\;ulcer\;index-treated\;index}{untreated\;ulcer\;index}\times \text{100}$$ (1)

#### 2.6.3 Assessment of Gastric Indices.

The stomach contents were collected, weighed, and centrifuged at 4000 rpm for 10 min to obtain the supernatant and pH estimations via a digital pH meter (Sartorius, Germany). Moreover, the total stomach acidity (TSA) for each stomach content was estimated by the addition of 1 mL of distilled water and 2–3 drops of phenolphthalein indicator. The NaOH (0.01 N) was used to titrate the final mixture solution until a pink color formed, which was followed by TSA calculation using Eq. (2):


$$\text{TSA}=\frac{\text{V}(\text{NaOH})\times \text{N}\times \text{100 mEq}/\text{L}}{\text{0}.\text{1}}$$ (2)

The gastric wall mucus was also evaluated for the Alcian blue binding (ABB) capacity following the previous procedure [22]. Briefly, the stomach’s glandular portions were dissected and deepened in Alcian blue mixture (10 mL of 0.1% w/v) for 2 h. The sucrose solution (10 mL, 0.25 M) was employed for the removal of excess dye; which is followed by the addition of 10 mL of 0.5 M magnesium chloride for 30 min. Furthermore, 4 mL of diethyl ether was mixed with plant extracts on an electric shaker, incubated for 2 min, then the mixture was centrifuged at 4000 rpm for 10 min, and the absorbance of the mucus fluid was measured at 580 nm. The ABB potentials were determined and presented as mg/gram of the glandular gastric tissues [19].

#### 2.6.4 Histological analysis.

The stomach samples were buffer-washed, sliced into small pieces (1–2 cm), and then fixed with 10% (v/v) paraformaldehyde [7]. After a fully automated fixation procedure, the gastric tissue was paraffinized and processed under a microtome for obtaining small sections of 5 –m gastric tissue. The gastric sections were placed on glass slides for different staining procedures using H & E, and PAS stains as detailed previously. After fully drying the slides in the oven, the slides were screened under a light microscope for the histopathological alterations (epithelial tissue injury, atrophy (loss of gastric glands), inflammatory cell infiltration, submucosal edema/penetration, congested areas, gastric lesion/hemorrhage, and deep tissue erosion) and mucopolysaccharide contents.

#### 2.6.5 Immunohistochemical.

The gastric tissues were examined for the amount of immunohistochemical proteins, including Bax and Bcl-2, to determine the level of apoptotic action. The gastric small sections were paraffinized, incubated with % hydrogen peroxide for 10 minutes, and then blocked with BCA solution for 30 minutes. The tissue samples were probed with anti-Bax (1:500 dilution, Solarbio, Wuhan, China) and anti-Bcl-2 (1:500 dilution, Solarbio, Wuhan, China) for 12 hours at 37 °C. The samples were then incubated with peroxidase-conjugated secondary antibody for 30 min at room temperature and screened via the SABC kit (Boster Biological Technology, Wuhan, China). The brown colored areas (immunopositive cells) under the microscope were considered as areas with P53 and Bcl-2 expressed cells, and the optical density was calculated by Image J analysis software via seven fields of each stomach particle [23].

#### 2.6.6 Antioxidants of tissue homogenates.

The gastric tissues were buffer-washed and homogenized via a Teflon homogenizer, which is followed by centrifugation of the mixture at 4500 rpm (15 min, 4 °C). The separated tissue supernatant was examined for the SOD, CAT, and GPx contents as well as intragastric MDA levels via purchasable ELISA kits from Solarbio, Wuhan, China [24].

#### 2.6.7 Inflammatory cytokines.

The collected amount of blood samples from animals was immediately transferred into labs, and after clotting and centrifugation at 4500 rpm for 50 min, the serum was evaluated for the amount of TNF-α, IL-6, and IL-10 cytokines using purchasable ELISA kits from Solarbio, Wuhan, China [25].

### 2.7 Statistics

Continuous variables between experimental groups are shown as mean ± standard error of mean (SEM). Statistical procedure conducted via one-way analysis of variance (ANOVA) followed by Tukey–Kramer as a post hoc test, as well as Kruskal–Wallis test and Dunn post-test were employed for calculating ulcer index. All statistical analyses and figure designs were possible using GraphPad Prism software version 9.01 (Inc., La Jolla, CA, USA).

## 3. Results

### 3.1 Phytoconstituents

**Fig 2:**
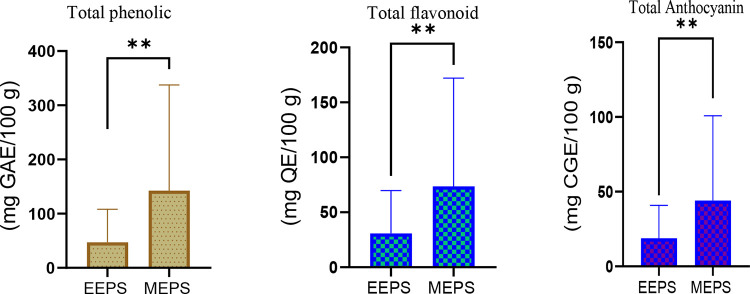
Phytochemical profiles of ethanolic and methanolic extracts of *P. syriaca* (EEPS and MEPS, respectively). The total phenolic compounds were superior to flavonoid and anthocyanin contents, while methanolic extracts showed higher phytoconstituents than ethanolic partitioning. Values are expressed as mean ± SEM of triplicates. **, P > 0.01.

The initial phytochemical characterization unveiled increased total phenolic (142.55 mg GAE/100 g extract), flavonoid (73.65 mg QE/100 g), and anthocyanin (44.05 mg CGE/100 g) contents in the methanolic extracts of *P. syriaca* than those (47.2 mg GAE/100 g, 30.8 mg QE/100 g, and 18.85 mg CGE/100 g, respectively) of its ethanolic extracts (Fig 2). The total phenolic content was the predominant phytoconstituent of *P. syriaca* fruits regardless of which solvent was used. As the results indicated, the methanolic extracts were richer in terms of all three estimated phytoconstituents; therefore, the methanolic extracts were prepared in large quantities for the later acute toxicity and gastroprotective trials.

### 3.2 Acute toxicity

**Fig 3:**
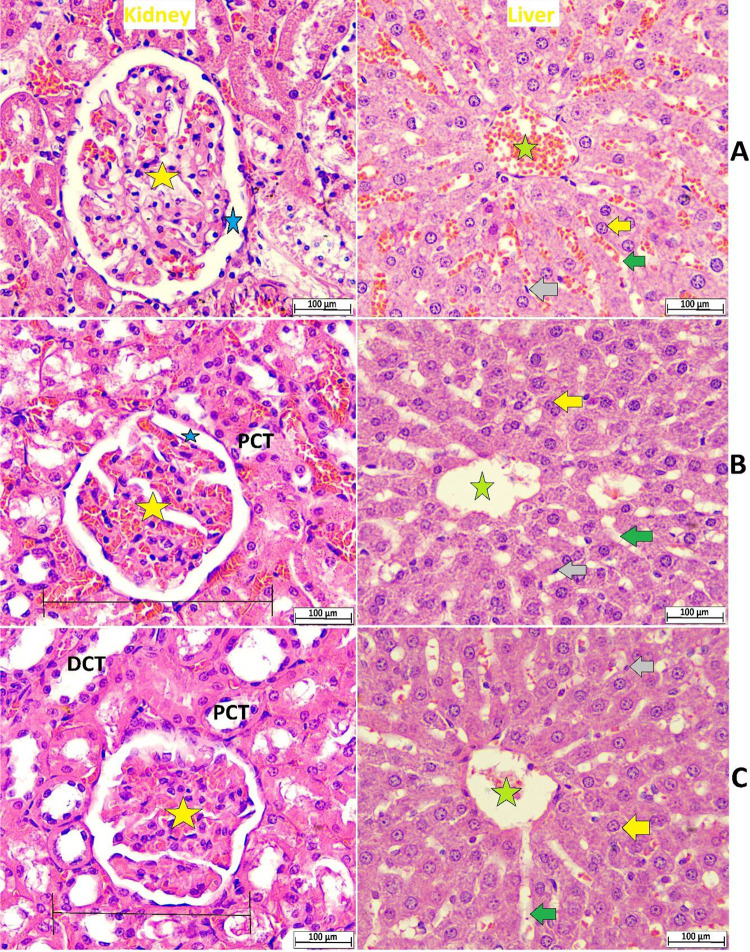
Microscopic appearance of dissected organs from rats treated with either normal saline (A), 2 g/kg MEPS (B), or 5 g/kg MEPS (C). green star, central vein; yellow arrow, sheets of hepatocyte; gray arrow, Kupffer cells; green arrow, sinusoids; yellow star, glomerulus; DCT, distal convoluted tubules; blue star, bowman’s space; PCT, proximal convoluted tubules; black line, glomerulus with bowmen’s capsule (H & E staining, 40x).

The single dose administration of chosen doses (2 and 5 g/kg MEPS) did not result in any toxicity incidence or physiological abnormalities during or after the trial. Supplemented rats did not exhibit clinical or abnormal behaviors or symptoms such as salivation, tremors, diarrhea, skin/eye color changes, Light/sound sensitivity, Painful feelings, or neurovegetative reactions. A regular checkup did not find any irregularities in the sleep, locomotion, or food/water intake in MEPS intragastric-treated rats. The histopathological evaluation of dissected organs showed comparable tissue architecture between normal control and MEPS-supplemented rats without any tissue disruption signs such as necrosis, hemorrhage, tubular necrosis, or lesions (Fig 3). Moreover, relevant biochemical parameters, including liver and kidney tests, were not significantly varied between normal control and MEPS-supplemented rats (available on request). The above data evidenced an increased safety margin of MEPS with NOAEL levels determined as 5 g/kg MEPS.

### 3.3 Assessment of gastroprotective activity

#### 3.3.1 Macroscopic views.

**Fig 4:**
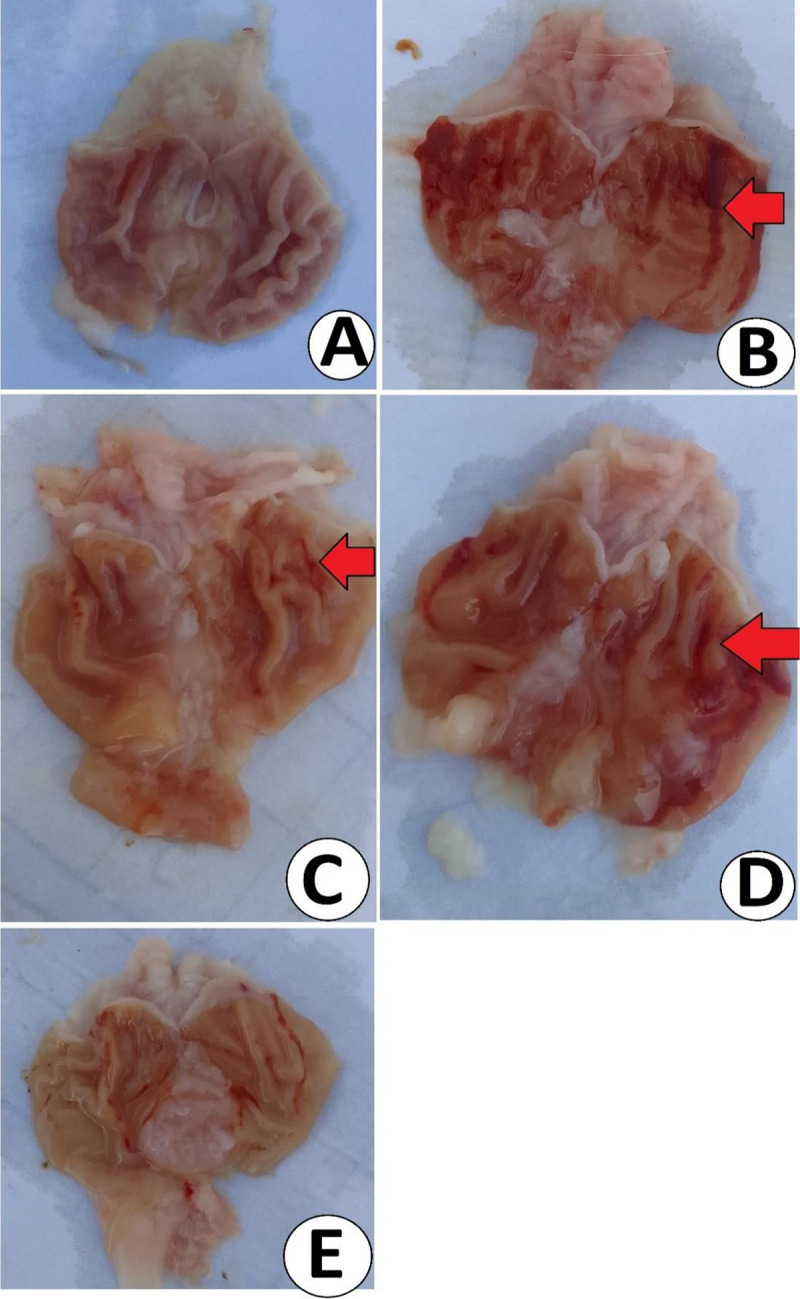
Gastric gross views of rats receiving either physiological saline (A); physiological saline+ absolute ethanol (B); 30 mg/kg lansoprazole+ absolute ethanol (C); 250 mg/kg MEPS + absolute ethanol (D); 500 mg/kg MEPS + absolute ethanol (E). Ulcer controls exhibited increased redness areas, hemorrhage (bleeding), erosions (shallow breaks on the surface mucosa), and erythema (redness). Such mucosal tissue injury was ameliorated in rats pretreated with lansoprazole or MEPS in a dose-related manner.

The gross views of stomach organs are presented in Fig 4. Ethanol oral delivery caused noticeable mucosal hemorrhage (dark/red areas) extending as a thick line across the gastric axis, severe gastric tissue injury, deeper sores, shallow breaks in the lining, and bleeding, which were clearly observed in ulcer control rats. Rats held as normal control rats were shown with clear pink mucosal layers with numerous gastric folds. Lansoprazole or MEPS pretreatment attenuated ethanol-mediated stomach injury, shown by fewer hemorrhagic areas, pinpoint red spots, less swollen/bleeding areas, and more flattened as well as higher mucosal folds relative to ulcer controls. Wild pear pretreatment at 500 mg/kg MEPS displayed a remarkable reduction in the lesion/ulcer indices, less mucosal injury, as well as normal tissue morphology and higher gastric rugae folds compared to ulcer control and low-dose-treated rats.

#### 3.3.2 Effect of MEPS on gastric defense factors and ulcer estimations.

The intragastric administration of ethanol caused noticeable weakening of gastric defense systems (gastric pH, mucus secretion, and mucopolysaccharide). The untreated group displayed an increased ulcer index compared to the normal control rats, which was also higher than that of lansoprazole or MEPS (250 and 500 mg/kg)-treated rats. The estimated ulcer protective index was significantly up-regulated in rats pre-ingested with lansoprazole or MEPS (250 and 500 mg/kg) treated (66.80%, 22.13%, and 50.40%, respectively), using the ulcer index of ulcer controls as a positive standard. Moreover, TAS was higher, and the Alcian blue binding (ABB) potentials were lower in the ulcer control rats, which further deteriorated gastric mucosal injury. In contrast, MEPS pretreatment restored stomach acidity and enhanced ABB potentials, indicated by TSA (21.23, 72.34, 31.50, 44.20, and 39.45 mEq/L/100 g) and higher Alcian blue binding potentials (721.2, 165.3, 354.3, and 483.2 mg/g) compared to untreated rats (Table 1).

**Table 1:** Effect of MEPS on some stomach contents.

Animal group	Ulcer index	Protective index %	Mucus weight g	pH	TSA (mEq/L/100 g)	mg Alcian blue/g tissues
**A**	Nil		3.10 ± 0.33^a^	6.83 ± 0.34^a^	21.23 ± 2.4^a^	721.2 ± 3.7^a^
**B**	4.88 (4–5.5)^d^	_	0.53 ± 0.62^d^	4.32 ± 0.54^b^	72.34 ± 3.4^d^	165.3 ± 4.1^e^
**C**	1.62 (1.1–2.2)^a^	66.80^c^	2.19 ± 0.59^b^	6.45 ± 0.50^a^	31.50 ± 3.4^b^	610.2 ± 4.4^b^
**D**	3.8 (3–4.4)^c^	22.13^a^	1.56 ± 0.54^c^	5.23 ± 0.43^d^	44.20 ± 4.2^c^	354.3 ± 4.9^d^
**E**	2.42 (2.1–3.2)^b^	50.40^b^	1.32 ± 0.74^b^	6.50 ± 0.73^a^	39.45 ± 2.7^c^	483.2 ± 5.2^c^

Different superscripts on numbers within the same column indicate significance P > 0.05. Rats receiving either physiological saline (A); physiological saline + absolute ethanol (B); 30 mg/kg lansoprazole + absolute ethanol (C); 250 mg/kg MEPS + absolute ethanol (D); 500 mg/kg MEPS + absolute ethanol (E).

The gastric pH and the produced mucus in the stomachs were found to vary significantly between experimental rats. The ulcer control exhibited a decrease in mucus content (82.90% decrease) and gastric pH relative to normal controls, which facilitated further mucosal penetrations. While lansoprazole or MEPS (250 and 500 mg/kg) pretreatment increased mucus content by 313.20%, 194.33%, 149.05%, respectively, and gastric pH by 49.30%, 21.06%, 50.46%, respectively, using values of ulcer controls as positive standards (Table 1).

#### 3.3.3 Histopathological examination using H&E stain.

**Fig 5:**
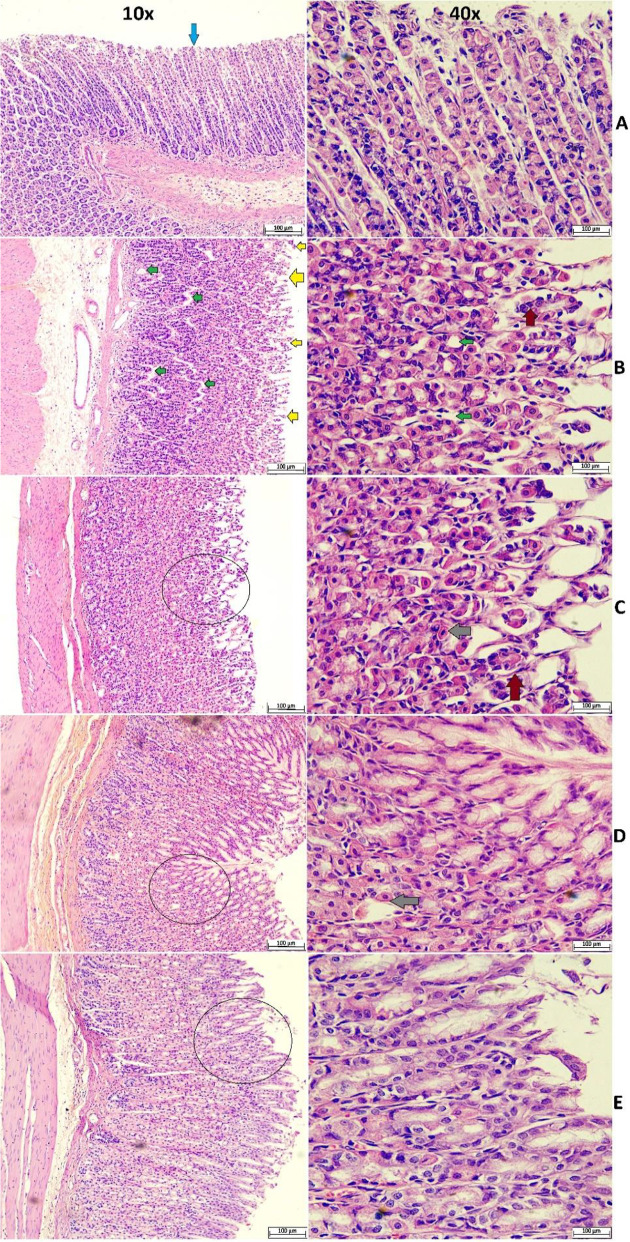
Microscopic structure (10x and 40x) of Gastric tissue for rats receiving either physiological saline (A); physiological saline+ absolute ethanol (B); 30 mg/kg lansoprazole+ absolute ethanol (C); 250 mg/kg MEPS + absolute ethanol (D); 500 mg/kg MEPS + absolute ethanol (E). Ulcer control exhibited epithelial disruption and detachment (yellow arrow), increased vacuoles in the mucosal cell lining (green arrow), and increased inflammatory infiltration (gray arrow). MEPS treatment preserved mucosal integrity and reduced submucosal edema and inflammatory cell infiltration. blue arrow, intact epithelium; Black circle, area of mucosal erosion/ulceration; gray arrow, inflammatory cells; brown arrow, apoptosis injury.

The histological evaluation of dissected stomachs from normal controls showed normal tissue layers in the mucosa, serosa, muscularis mucosa/serosa, and submucosa layers. Ulcer controls exhibited pronounced ulcer/lesion areas with increased mucosal penetrations, focal ulceration, hemorrhagic areas, and severe epithelial tissue disruptions. Moreover, the gastric tissues of ulcer-induced controls were characterized by clear, large edema in the submucosal layer as well as increased inflammatory cell infiltration. In contrast, gastric specimens from lansoprazole-treated or (250 and 500 mg/kg)-treated rats presented renewal of the stomach mucosa in a dose-related fashion. Rats receiving 250 mg/kg exhibited intact mucosa with diffuse leukocyte/inflammatory cells infiltration as well as smaller submucosal edema and blood vessels congestion, while the higher dose (500 mg/kg)-treated rats were highlighted with more intact mucosa, significantly less submucosal edema, fewer cellular necrosis, and reduced blood vessels congestion/inflammatory cells cand few focal inflammatory cell infiltrations compared to low dose and ulcer inducted rats (Fig 5). The gastric tissue recovery, including the reappearance of the epithelial/stromal layers as well as submucosal edema inhibition, was found to be comparable between 500 mg/kg and lansoprazole-treated rats.

**Fig 6:**
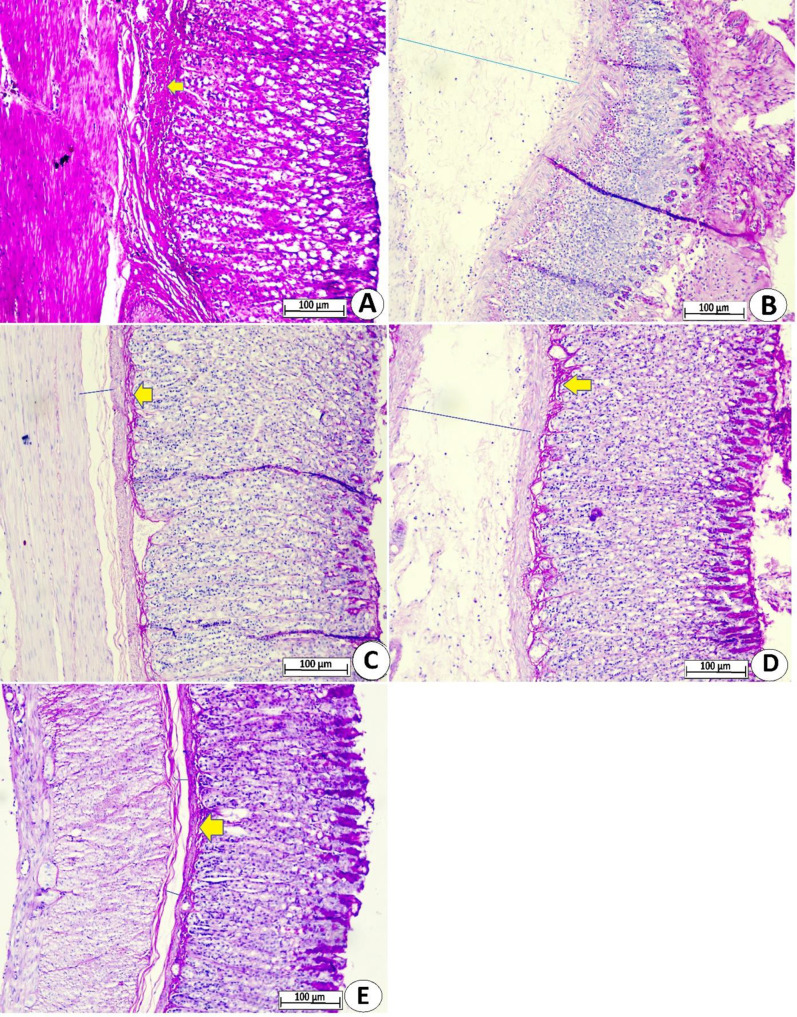
Microscopic gastric tissue views (10x) showing different levels of PAS in rats physiological saline(A); physiological saline+ absolute ethanol (B); 30 mg/kg lansoprazole+ absolute ethanol (C); 250 mg/kg MEPS + absolute ethanol (D); 500 mg/kg MEPS + absolute ethanol (E). MEPS-treated rats showed increased expression of PAS stains in gastric tissues, denoting positive modulation of mucopolysaccharides/glycoproteins in gastric tissues that act as a physical barrier against ethanol-mediated gastric mucosal injury.

The histopathological investigation showed different levels of PAS stains in gastric tissues, indicating variations in the production of mucopolysaccharides and mucins between ulcer-inducing rats and pre-ingested lansoprazole or MEPS-treated rats. The gastric tissue of ulcer controls is presented with reduced intensity of PAS stains (very light magenta areas), denoting a suppressed production of mucus by mucoid cells of gastric glands, all of which made the mucosa layer more exposed to ethanol-mediated tissue penetration. In contrast, lansoprazole or MEPS (250 and 500 mg/kg) improved gastric gland potentials and mucoid cell secretions, resulting in increased production of alkaline mucopolysaccharides/glycoproteins that maintain gastric pH and prevent gastric mucosa erosion/penetrations by aggressive factors such as absolute ethanol (Fig 6).

#### 3.3.4 Expression of apoptotic proteins.

**Fig 7:**
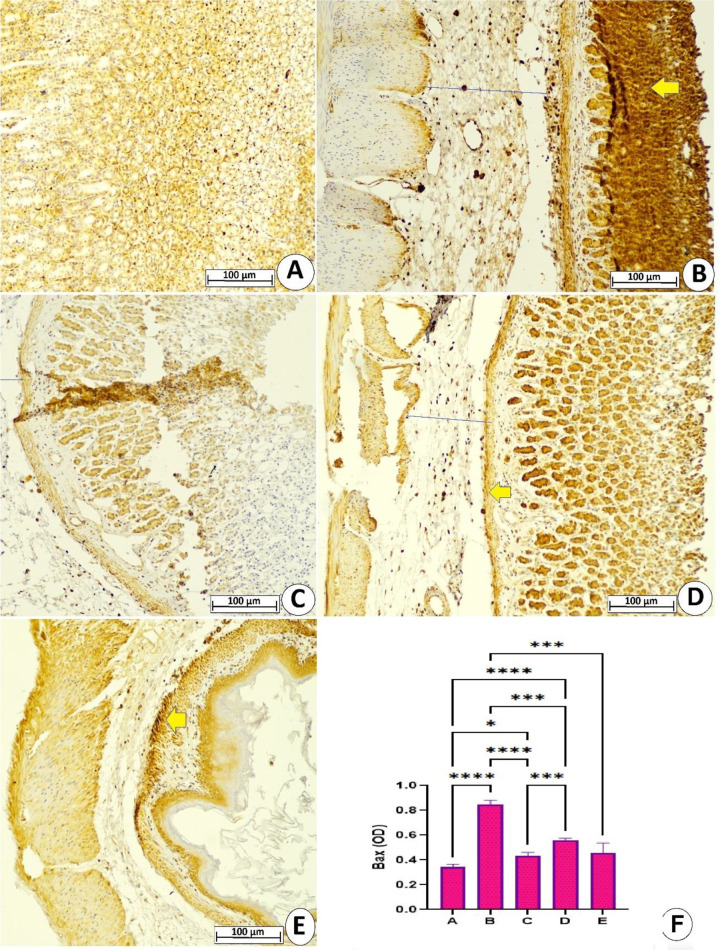
Microscopic gastric tissues (10x) presenting different levels of Bax proteins in rats receiving either physiological saline (A); physiological saline+ absolute ethanol (B); 30 mg/kg lansoprazole+ absolute ethanol (C); 250 mg/kg MEPS + absolute ethanol (D); 500 mg/kg MEPS + absolute ethanol (E). The ulcer control rats exhibited increased Bax protein (brown colored areas) in their stomachs, denoting elevated apoptotic actions that further deteriorate the stomach injuries. While lansoprazole or MEPS (250 and 500 mg/kg) administration reduced Bax protein expression, suppressing ethanol-mediated apoptotic tissue injury occurred. The optical density of Bax-expressing cells was significantly lower in lansoprazole and MEPS-supplemented rats relative to ulcer-inducing rats **(F)**. *, P > 0.05; ***, P > 0.001; ****, P > 0.0001.

**Fig 8:**
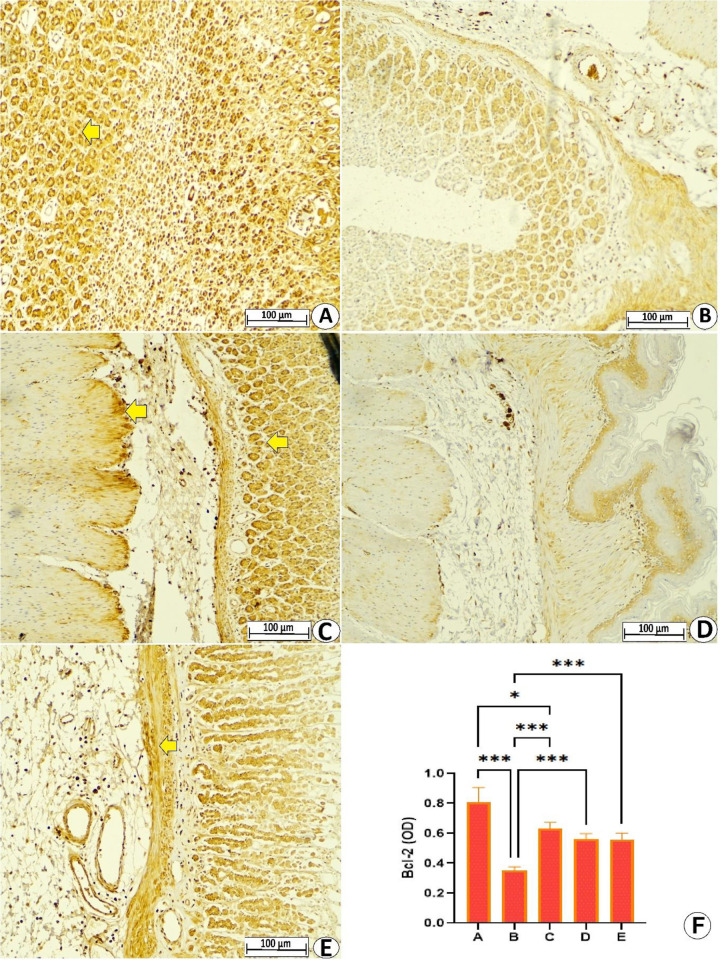
Microscopic gastric tissues (10x) presenting different levels of Bcl-2 proteins in rats receiving either physiological saline (A); physiological saline+ absolute ethanol (B); 30 mg/kg lansoprazole+ absolute ethanol (C); 250 mg/kg MEPS + absolute ethanol (D); 500 mg/kg MEPS + absolute ethanol (E). The ulcer control rats exhibited reduced Bcl-2 proteins (brown colored areas) in their stomachs, which enhanced apoptotic actions that further deteriorated the stomach injuries. While lansoprazole or MEPS (250 and 500 mg/kg) administration up-regulated Bcl-2 protein expression, suppressing ethanol-mediated apoptotic tissue injury and thus less gastric tissue damage. The optical density of Bcl-2-expressing cells was significantly higher in lansoprazole and supplemented rats relative to ulcer control rats **(F)**. *, P > 0.05; ***, P > 0.001.

Immunohistochemical results showed different levels of apoptotic proteins in the gastric tissues of rats exposed to ethanol and different pretreatments. Fig 7 and Fig 8 present reduced expression of Bax and Bcl-2 proteins in gastric tissues of normal control rats, indicating reduced or a lack of any apoptotic actions. The ulcer control rats showed increased pro-apoptotic Bax proteins and reduced cytoplasmic Bcl-2 protein expression. The results of immunoreactivity evaluation confirmed that ethanol ingestion caused a significant increase in optical density of Bax-expressing cells by 146.37% and notably reduced Bcl-2-expressing cells by 56.25% compared to normal control rats. In contrast, pretreatment with lansoprazole or MEPS (250 and 500 mg/kg) led to significant suppression of apoptotic actions, evidenced by reduced Bax proteins and increased Bcl-2 proteins in gastric tissues, with supremacy of the high dose in modulating expression of apoptotic proteins. The immunoreactivity calculation confirmed that lansoprazole or MEPS (250 and 500 mg/kg) decreased the optical density of Bax proteins by 49.41%, 35.29%, and 47.05%, respectively, while increasing the optical density of Bcl-2 expressing cells by 80%, 51.42%, and 60%, respectively, relative to values of ulcer control rats. The above data evidence significant anti-apoptotic potentials of MEPS in rats exposed to ethanol-mediated gastric cell death, which could be one of the mechanistic preventive ways of its anti-ulcer effects (Fig 7 and Fig 8).

#### 3.3.5 Effect of MEPS on oxidative stress.

**Fig 9:**
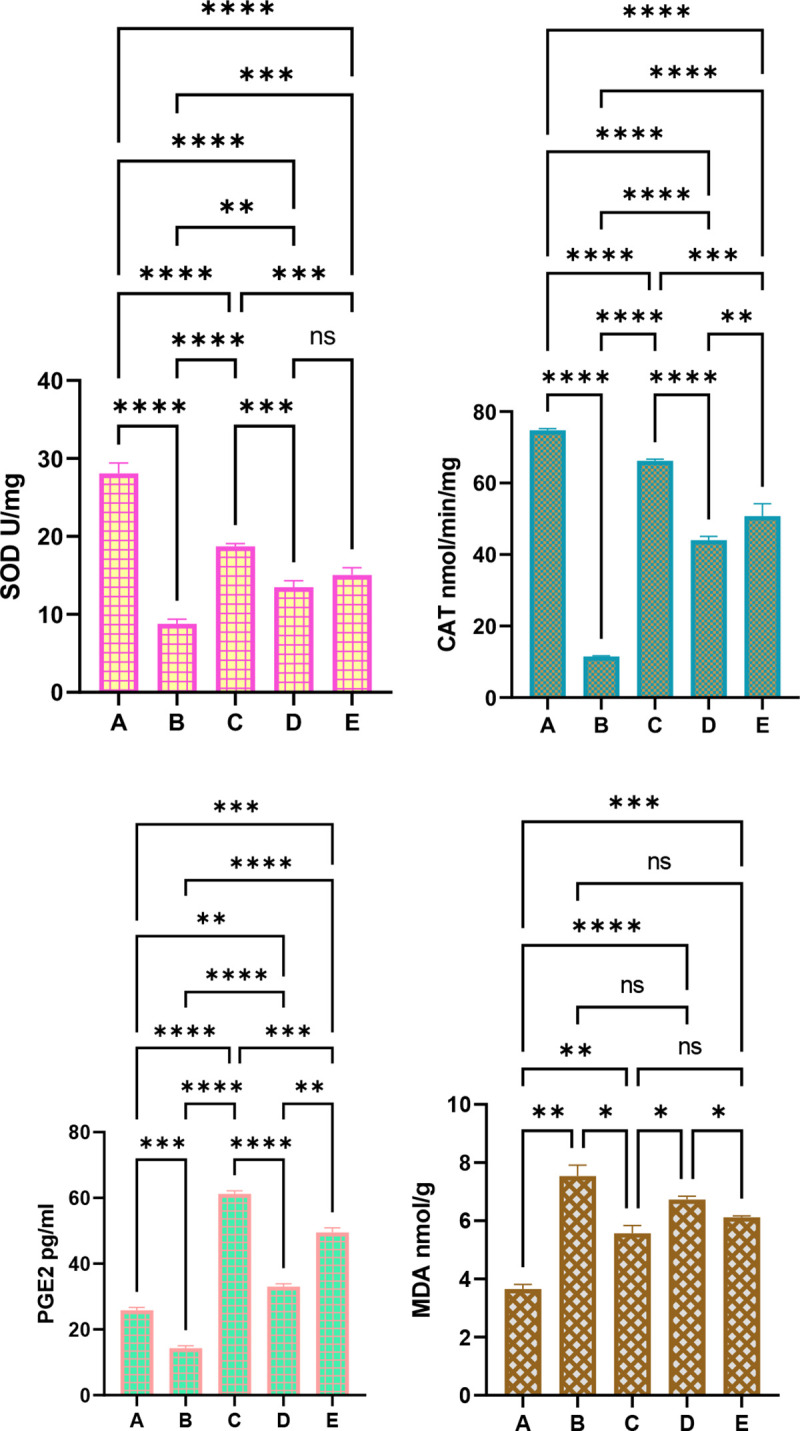
Concentration of MDA and antioxidants in gastric tissues of rats receiving either physiological saline (A); physiological saline+ absolute ethanol (B); 30 mg/kg lansoprazole+ absolute ethanol (C); 250 mg/kg MEPS + absolute ethanol (D); 500 mg/kg MEPS + absolute ethanol (E). The ulcer control rats exhibited severe ethanol-mediated oxidative tissue injury, denoted by up-regulated MDA and reduced antioxidants. While MEPS supplementation enhanced production of endogenous antioxidants that attenuated oxidative stress in gastric tissues exposed to ethanol-mediated ulceration, as indicated by significantly higher antioxidant enzymes and lower MDA index relative to ulcer controls. *, P > 0.05; **, P > 0.01; ***, P > 0.001; ****, P > 0.0001.

The gastric MDA levels and the endogenous antioxidants are evaluated to unveil the antioxidant potentials of MEPS in rats exposed to ethanol-mediated gastropathy (Fig 9). As a metabolic oxidative stress marker, the MDA levels were reasonably low, and the efficient antioxidants were found in normal control rats. The ulcer control rats showed reduced SOD (69.58%), CAT (84.61%), and PGE2 (45.82%) enzymes, as well as increased gastric MDA (106.30%) contents compared to normal control and other treated groups. While lansoprazole or MEPS (250 and 500 mg/kg) treatment suppressed ethanol-mediated oxidative tissue injury, which increased the SOD by 114.94%, 57.93%, 73.10%; CAT by 476.67%, 283.37%, 385.3%; PGx by 321.42%, 115.5%, and 246.4%; while reducing MDA levels by 26.16%, 10.75%, 18.72%, respectively. The above data indicate significant antioxidant potentials that ameliorated lipid peroxidation/oxidative tissue injuries provoked by ethanol intragastric delivery.

#### 3.3.6 Effect of MEPS on inflammation.

**Fig 10:**
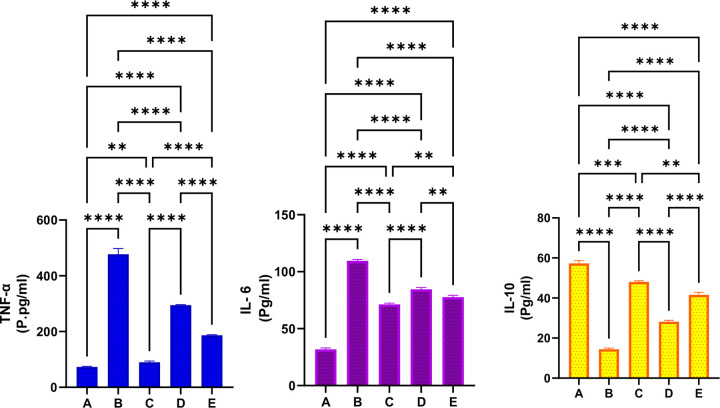
Concentration of inflammatory cytokines in serum of rats receiving either physiological saline (A); physiological saline+ absolute ethanol (B); 30 mg/kg lansoprazole+ absolute ethanol (C); 250 mg/kg MEPS + absolute ethanol (D); 500 mg/kg MEPS + absolute ethanol (E). Ethanol oral delivery provoked a significant inflammatory response that delayed ulcer healing. Noticeably, MEPS supplementation attenuated ethanol-mediated inflammation, indicated by significantly pro-inflammatory cytokines and up-regulated IL-10 cytokines, all of which shortened the inflammatory response that accelerated ulcer healing.

The intragastric ethanol administration caused a significant inflammatory response, shown by up-regulated TNF-α by 552.31% and IL-6 by 244.33%, while reducing serum IL-10 by 74.77% compared to normal control rats. In contrast, lansoprazole or MEPS (250 and 500 mg/kg) reduced TNF-α by 81.19%, 38.23%, 60.82% and IL-6 by 34.86%, 22.73%, 29.02% as well as increased IL-10 by 232.08%, 94.53%, 187.41%, respectively, relative to values of ulcer-induced rats. The present data estimations revealed significant anti-inflammatory potentials of MEPS, which shortened the inflammatory response and inflammatory cell infiltration, subsequently accelerating gastric ulcer healing (Fig 10).

## 4. Discussion

Phytoconstituents have gained renewed interest in the last few decades due to their increased therapeutic potential as well as fewer side effects [26]. The current study determined increased phenolic, anthocyanin, and flavonoid contents. The latter one is considered a large category of phenolic compounds with tremendous curative efficacy for various human disorders, and is among numerous valuable phytoconstituents of edible wild fruits. Accordingly, researchers reported increased total phenolic contents from Wild pear (*Pyrus eleagnifolia* Fruit), ranging from 42.79 to 119.14 mg GAE/100 g [27]. Some genotypes of P. syriaca have been highlighted as a rich source of ascorbic acid and total phenolics [28]. Kyahan et al. have shown tremendous amounts of phenolics and total flavonoids in *P. amygdaliformis*, *P. anatolica*, and P*. elaeagnifolia*, with Supremacy of *P. anatolica* extract (47.020 ± 1.652 mg GAE/g and 20.903 ± 0.025, respectively) [29]. Phytochemical profiling of 10 pear genotypes unveiled Phenolic (140–650 GAE mg/100 g), Anthocyanin (1.4–20.2 CGE mg/100 g), and ascorbic acids (mg/100 g) as major chemical contents of their methanolic extracts [30].

The toxicity incidence is the main drawback associated with the consumption of any plant-based ingredients with potential therapeutic efficacies [31]. In this context, the present study evaluated the acute toxicity effect of a single oral dose of 2 g/kg and 5 g/kg MEPS in male and female rats, which did not cause any lethality or physiological abnormalities; therefore, with an LD_50_ of 5000 mg/kg, it can be classified as category 5 of non-toxic ingredients in align with GHS thresholds for categorization [32]. To our best knowledge, this is the first safety evaluation of *P. syriaca*, however, Numerous studies have confirmed non-toxic effects of *Pyrus* fruits (pears) for most people, with few reports describing possible drawbacks such as carbohydrate metabolism disorders, including non-specific diarrhea, and irritable bowel syndrome in infants and children [33].

Gastric ulcers can be initiated by heavy alcoholism, disrupting physical barriers and penetrating the gastric mucosa, altogether forming gastric erosions as well as gastritis, which is recognized by submucosal edema, cellular exfoliation, inflammatory cell infiltration, and sub-epithelial hemorrhages. The gastric ulcer severity can be evaluated by lesion formats and histopathological alterations. Tremendous data support that traditional plants can have gastroprotective effects against aggressive factors such as ethanol by strengthening the gastric defense system, down-regulation of gastric acids, or increasing production of mucopolysaccharides/glycoproteins [20]. Thus, the present study was geared to unveil the anti-ulcerogenic effects of MEPS in ethanol-mediated ulceration in rats. In line with the literature data, our data showed that intragastric delivery provoked significant ulcer initiation and gastric tissue injury, while pretreatment with MEPS resisted ethanol-induced stomach injury. MEPS supplementation inhibited ulcer index and lowered structural tissue alterations. Microscopic screening confirmed gastroprotective effects of MEPS, denoted by fewer vacuolization, less tissue necrosis, and less epithelial injuries from the upper portion of fundic glands, and deep dilated inter-glandular spices. Such gastroprotective effects of MEPS is parallel with its phytoconstituents (phenolics, flavonoids, and anthocyanins), which were repeatedly highlighted as anti-ulcer ingredients/compounds [33,34]. In a similar line, Bobe et al. also reported significant gastroprotective effects of wild pear (*Pyrus ussuriensis* Maxim) extracts (250 and 500 mg/kg), which were mainly linked with its biological potentials to improve gastric defense factors as well as modulate several antioxidant and anti-inflammatory process, such increasing PGE_2_, while down-regulation cAMP, histamine/histamine receptor and H^+^/K^+^ ATPase pathway as well as muscarinic receptor [35].

The apoptosis process is considered a crucial pathological mechanism associated with ethanol-mediated gastric injury, which is mainly associated with mucosal inflammation. During stomach ulceration, severe gastric acid and increased ROS generation cause gastric mucosal epithelial apoptosis. Interestingly, mucosal cell loss is an outcome of mitochondrial apoptosis in the gastric mucosa, initiating gastric ulcers [19]. The intrinsic apoptosis pathway begins with the P53 gene, which provokes expression of pro-apoptotic proteins (Bax), P21, NOXA, and PUMA that, in turn, disrupt the mitochondrial membrane, cytochrome c release, and cell death occur after increased production of apoptosomes and caspase-3 activation. While Bcl-2 protein is considered an anti-apoptotic proteins that inhibit apoptotic pathways and provokes cell proliferation as well as angiogenesis [7]. In this context, the present study showed that ethanol ingestion provoked apoptosis-mediated gastric injury, evidenced by positive modulation of Bax proteins in gastric tissues. MEPS supplementation reduced apoptotic actions (decreased Bax and increased Bcl-2) in a dose-related manner. These outcomes suggest that MEPS pretreatment ameliorated the initiation of the mitochondrial-apoptotic mechanisms of stomach mucosal cells, thus presenting an anti-apoptosis strategy to resist gastric ulcer injury. Such Anti-apoptotic actions of MEPS could be related to its detected phytoconstituents (phenolics, flavonoids, and anthocyanins), which were repeatedly reported as anti-apoptotic compounds [36,37]. Accordingly, researchers have shown significant modulatory potentials of *Pyrus ussuriensis* Maxim (250 and 500 mg/kg body weight) on immunohistochemical (intraepithelial lymphocyte infiltration), which was mainly linked with its phytoconstituents [35].

Ethanol metabolic byproducts, including acetaldehyde and acetate, can provoke oxidative stress through stimulation of cytochrome P450 2E1, and NADH-related pathways, generating harmful reactive oxygen species (hydroperoxide and superoxide anion). In case of alcohol overuse, ROS upregulation causes the buildup of fatty acids and other hazardous agents such as MDA and 4-hydroxynonenal, as seen in our ulcer control groups. MEPS pretreatment ameliorated ethanol-mediated oxidative stress tissue injury, indicated by lower antioxidant contents (SOD, CAT, and PGE2) and lipid peroxidation byproducts. Such antioxidant potentials could be linked with its phytoconstituents (flavonoids, phenolics, anthocyanins), which are parallel with the previous findings. In a similar line, gastroprotective effects of *P. ussuriensis* wild pear extracts (250 and 500 mg/kg) have been linked with its phytoconstituents’ antioxidant potentials, which were further confirmed by in vitro assays (DPPH and ABTS) [35]. Accordingly, previous studies evidenced significant actions of Pyrus extracts or their phytoconstituents mediated by their modulatory actions on Keap1-Nrf2 (for endogenous antioxidants) and other oxidative stress-related pathways, including the MAPK and PI3K/AKT pathways that are important players of cell survival/death [14,38,39].

**Fig 11:**
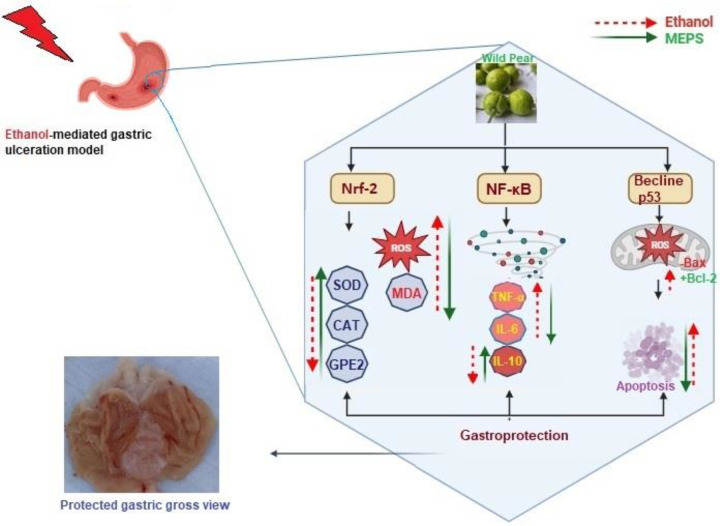
Summary of the possible underlying gastroprotective pathways modulated by MEPS in ethanol-mediated gastropathy in rats. Ethanol oral delivery reduced the expression of Nrf2, ameliorated the production of SOD, GSH, and HO-1, and up-regulated MDA levels. Conversely, MEPS supplementation down-regulated NF-KB expression and its inflammatory factors (TNF-α and IL-6). Pretreatment with MEPS attenuated ethanol-mediated autophagy by reducing Bax proteins and increasing Bcl-2 proteins. MEPS delivery resisted ethanol-mediated oxidative stress pathways, indicated by activated Nrf2 pathways and their antioxidant mediators SOD, CAT, PGE_2_, and lowered MDA level.

Inflammation is an inevitable cellular process involved in gastric ulcer initiation, which exacerbates the mucosal injury through leukocyte infiltration as well as macrophage migration to the ulcer site and its surroundings. Alcohol can provoke inflammation through stimulation of the NF-κB pathway, which is a vital key player in the development of gastric ulcers [40]. During the normal physiological state, NF-κB is kept inactive in the cytoplasm through the formation of a complex protein alongside the inhibitory IκBα protein. Once the stomach is exposed to aggressive factors such as ethanol, IκBα phosphorylation takes place with subsequent degradation of the proteasome; hence, activated NF-κB moves into the nucleus and positively modulates pro-inflammatory genes responsible for cyclooxygenase (COX)-2, TNF-α, and IL-6 expressions. Thus, NF-κB p65 subunit is recognized as an indicator for NF-κB activation and as a perfect target molecule for a therapeutic approach for the attenuation of the gastric ulcer. Moreover, COX-2 is a provoking isoenzyme that mediates the conversion of arachidonic acid to prostaglandins, which is considered a vital inflammatory mediator that is activated from a quiescent state [23]. In a similar line, the present ethanol ingestion up-regulated inflammatory responses, indicated by increased TNF and IL-6 cytokine expression, while lowering IL-6 levels. Conversely, MEPS pretreatment ameliorated ethanol-mediated inflammation, which could be linked with its detected phytochemicals (phenolics, flavonoids, and anthocyanins) that reduced expression of inflammation-related pathways (NF-κB) and decreased pro-inflammatory secretions (TNF-α and IL-6). Similarly, extracts of wild pear (*Pyrus ussuriensis*) were presented as strong anti-inflammatory agents supported by enriched phytochemical contents (hydroxycinnamic acids, flavanols, flavonols, flavones, hydroquinones, and anthocyanins) [41]. Accordingly, phenolic and flavonoid compositions of EtOAc extracts (200 and 400 mg/kg) of *Pyrus bretschneideri* Rehd. has been linked with its significant anti-inflammatory actions against xylene-mediated ear edema [42]. The possible mechanisms underlying the gastroprotective potentials of MEPS are shown in Fig 11.

## 5. Conclusion

The outcomes present the spectrophotometric-based phytochemical exploration of MEPS, highlighting phenolic compounds as dominant phytoconstituents in these wild fruit extracts. Besides, the present data present the first new insights into acute toxicity and gastroprotective effects of this wild *Pyrus* fruit in ethanol-mediated gastric ulcers. The results indicated that MEPS possesses the promising bio-potentials that are partially related to its antioxidant through up-regulating SOD, CAT, GPx, and reducing MDA levels, as well as its anti-inflammatory actions through suppressing pro-inflammatory cytokine release (TNF-α and IL-6). Moreover, MEPS supplementation led to down-regulation of the apoptotic process in gastric tissues exposed to absolute ethanol, shown by marked Bax depression and enhanced Bcl-2 expression comparable to the lansoprazole reference drug. Therefore, the findings present *P. pyrus* as a rich source of natural products and evidence its safe ingestion as well as its gastroprotective potentials, which should be further considered for their in-depth molecular identification and isolation before being employed in clinical trials as new gastric therapeutics.

## Supporting information

S1 File For Fig. 2 https://github.com/ahmedabuljabbar-wq/s1.git. (XLSX)

S2 File For Fig. 7 https://github.com/ahmedabuljabbar-wq/s2.git. (DOCX)

S3 File For Fig. 8 https://github.com/ahmedabuljabbar-wq/s3.git. (DOCX)

S4 File Fig. 9 https://github.com/ahmedabuljabbar-wq/s4.git. (DOCX)

S5 File For Fig. 10 https://github.com/ahmedabuljabbar-wq/s5.git. (DOCX)
