# Supplementary material for: Chemical constituents and anti-ulcer effects of a wild pear (Pyrus syriaca Boiss.): Phytochemical, histopathological and apoptotic approaches
Source: PLoS One. 2026 Apr 2;21(4):e0344660. doi: 10.1371/journal.pone.0344660 (PMC13046164; doi:10.1371/journal.pone.0344660)
Supplement: S2 File — (DOCX) [file pone.0344660.s002.docx]

| Total phenolic | R1 | R2 | R3 |
| --- | --- | --- | --- |
| EEPS | 47.3 | 45.9 | 48.2 |
| MEPS | 144.05 | 142.2 | 141.4 |

| Total Flavonoid | R1 | R2 | R3 |
| --- | --- | --- | --- |
| EEPS | 30.1 | 29.8 | 30.36 |
| MEPS | 74.62 | 72.83 | 73.5 |

| Total Anthocyanin | R1 | R2 | R3 |
| --- | --- | --- | --- |
| EEPS | 19.23 | 18.54 | 18.78 |
| MEPS | 43.02 | 43.9 | 45.25 |
