# Supplementary material for: Chemical constituents and anti-ulcer effects of a wild pear (Pyrus syriaca Boiss.): Phytochemical, histopathological and apoptotic approaches
Source: PLoS One. 2026 Apr 2;21(4):e0344660. doi: 10.1371/journal.pone.0344660 (PMC13046164; doi:10.1371/journal.pone.0344660)
Supplement: S3 File — (DOCX) [file pone.0344660.s003.docx]

| Replicates Bcl-2 | R1 | R2 | R3 | R4 | R5 | R6 |
| --- | --- | --- | --- | --- | --- | --- |
| A | 0.74 | 0.84 | 0.89 | 0.82 | 0.65 | 0.91 |
| B | 0.31 | 0.34 | 0.37 | 0.35 | 0.38 | 0.36 |
| C | 0.63 | 0.62 | 0.71 | 0.64 | 0.60 | 0.59 |
| D | 0.54 | 0.59 | 0.48 | 0.45 | 0.61 | 0.52 |
| E | 0.61 | 0.49 | 0.58 | 0.54 | 0.56 | 0.58 |
