# Supplementary material for: Chemical constituents and anti-ulcer effects of a wild pear (Pyrus syriaca Boiss.): Phytochemical, histopathological and apoptotic approaches
Source: PLoS One. 2026 Apr 2;21(4):e0344660. doi: 10.1371/journal.pone.0344660 (PMC13046164; doi:10.1371/journal.pone.0344660)
Supplement: S4 File — (DOCX) [file pone.0344660.s004.docx]

| Replicates SOD | R1 | R2 | R3 | R4 | R5 | R6 |
| --- | --- | --- | --- | --- | --- | --- |
| A | 28.3 | 29.1 | 27.6 | 25.7 | 29.4 | 28.45 |
| B | 8.1 | 8.34 | 9.4 | 9.54 | 8.5 | 8.86 |
| C | 18.34 | 18.49 | 18.5 | 18.59 | 19.3 | 19 |
| D | 14.5 | 14.3 | 13.2 | 12.7 | 12.43 | 13.7 |
| E | 15.6 | 14.3 | 13.9 | 14.8 | 15.34 | 16.4 |

| Replicates CAT | R1 | R2 | R3 | R4 | R5 | R6 |
| --- | --- | --- | --- | --- | --- | --- |
| A | 74.3 | 74.28 | 74.58 | 75.39 | 75.41 | 74.5 |
| B | 11.2 | 11.4 | 11.5 | 11.5 | 11.8 | 11.56 |
| C | 65.4 | 66.5 | 66.38 | 66.49 | 66.3 | 66.47 |
| D | 43.2 | 44.3 | 44.5 | 42.4 | 45.4 | 44.48 |
| E | 55.1 | 46.02 | 48.37 | 52.4 | 53.51 | 49.22 |

| Replicates PGE2 | R1 | R2 | R3 | R4 | R5 | R6 |
| --- | --- | --- | --- | --- | --- | --- |
| A | 25.3 | 30 | 24.3 | 25 | 25 | 25.43 |
| B | 13 | 15.34 | 15.9 | 16.4 | 12.8 | 12.45 |
| C | 59.3 | 58.4 | 61.2 | 63.2 | 64.2 | 61.29 |
| D | 31.45 | 34.87 | 34.5 | 35.4 | 31.89 | 30.1 |
| E | 51.2 | 43.28 | 48.32 | 50.84 | 53.2 | 50.32 |

| Replicates MDA | R1 | R2 | R3 | R4 | R5 | R6 |
| --- | --- | --- | --- | --- | --- | --- |
| A | 4.3 | 3.3 | 3.84 | 3.7 | 3.25 | 3.51 |
| B | 8.3 | 8.73 | 6.5 | 7.5 | 7.7 | 6.5 |
| C | 5.6 | 6.6 | 5.74 | 5.4 | 4.5 | 5.56 |
| D | 6.95 | 6.84 | 6.8 | 6.94 | 6.18 | 6.65 |
| E | 6 | 6.11 | 6.32 | 6.21 | 6.05 | 6.04 |
