# Supplementary material for: Chemical constituents and anti-ulcer effects of a wild pear (Pyrus syriaca Boiss.): Phytochemical, histopathological and apoptotic approaches
Source: PLoS One. 2026 Apr 2;21(4):e0344660. doi: 10.1371/journal.pone.0344660 (PMC13046164; doi:10.1371/journal.pone.0344660)
Supplement: S5 File — (DOCX) [file pone.0344660.s005.docx]

| Replicates TNF-α | R1 | R2 | R3 | R4 | R5 | R6 |
| --- | --- | --- | --- | --- | --- | --- |
| A | 76 | 73 | 71 | 71 | 73 | 75 |
| B | 496 | 455.2 | 485.6 | 468.4 | 456 | 502.3 |
| C | 88.3 | 81.3 | 91.2 | 92.3 | 94.3 | 91.2 |
| D | 295.4 | 294 | 293.4 | 294.5 | 298.1 | 293.4 |
| E | 188.2 | 184.3 | 187.3 | 185.4 | 189.3 | 187.3 |

| Replicates IL-6 | R1 | R2 | R3 | R4 | R5 | R6 |
| --- | --- | --- | --- | --- | --- | --- |
| A | 32.1 | 31.8 | 32.3 | 33.2 | 29.3 | 32.1 |
| B | 110.2 | 109.3 | 107.3 | 109.8 | 111.2 | 109.3 |
| C | 72.3 | 73.1 | 70.1 | 70.3 | 71.3 | 70.8 |
| D | 84.5 | 85.9 | 83.4 | 82.3 | 86.8 | 84.7 |
| E | 75.4 | 79.4 | 78.5 | 79.3 | 76.4 | 77.3 |

| Replicates IL-10 | R1 | R2 | R3 | R4 | R5 | R6 |
| --- | --- | --- | --- | --- | --- | --- |
| A | 55.9 | 56.3 | 56.7 | 58.3 | 57.3 | 59.4 |
| B | 14.8 | 14.3 | 13.97 | 15.3 | 13.7 | 14.7 |
| C | 47.3 | 48.2 | 49.1 | 47.5 | 48.2 | 47.8 |
| D | 28.3 | 28.9 | 27.4 | 27.8 | 27.3 | 29.1 |
| E | 41.8 | 41.3 | 40.73 | 43.9 | 41.5 | 40.12 |
